# Efficacy of 0.5% Tretinoin in the Treatment of Equine Aural Plaques

**DOI:** 10.1111/vde.70090

**Published:** 2026-05-29

**Authors:** Felipe Sperandio de Mattos, Lissandro Gonçalves Conceição, Ytalo Galinari Henrique Schuartz, Vitória Régia Melo Silva, Cintia Fernandes Fidélis, Fernanda Campos Mansur, Cristian Silva Teixeira, Yame Fabres Robaina Sancler‐Silva, Jose Paes Oliveira‐Filho, Alexandre Secorun Borges, Cristiana Raach Bromberger, José Dantas Ribeiro Filho, Marcel Ferreira Bastos Avanza, José Ricardo Barboza Silva, Raffaella Bertoni Cavalcanti Teixeira

**Affiliations:** ^1^ Departament of Veterinary Medicine Federal University of Viçosa (UFV) Viçosa Minas Gerais Brazil; ^2^ FAMINAS University Center Muriaé Minas Gerais Brazil; ^3^ Departament of Animal Science Federal University of Viçosa (UFV) Viçosa Minas Gerais Brazil; ^4^ Department of Veterinary Clinical Science São Paulo State University (Unesp), school of Veterinary Medicine and Animal Science Botucatu Brazil

**Keywords:** horses, imiquimod, papillomavirus

## Abstract

**Background:**

Equine aural plaques are a benign form of auricular papillomatosis caused by equine papillomavirus. Despite the efficacy of 5% imiquimod cream, a marked local reaction is frequently observed during therapy, often requiring sedation before application.

**Hypothesis/Objectives:**

The aim of this study was to evaluate the efficacy and potential adverse effects of 0.5% topical tretinoin in the treatment of equine aural plaques. We hypothesised that tretinoin would be effective, with fewer adverse effects compared to imiquimod.

**Animals:**

The study included 10 horses of both sexes, aged between 7 months and 15 years, diagnosed with unilateral or bilateral aural plaques (totalling 17 ears).

**Materials and Methods:**

This was a controlled clinical trial. Three horses underwent a pilot study, in which only one ear per animal was treated to allow for intra‐animal comparison, while the remaining seven horses received treatment in both ears. Lesions were classified into three groups based on the percentage of the inner auricular surface affected. Treatment was performed with 0.5% topical tretinoin once daily until lesion stabilisation or resolution. Lesion characteristics, treatment response, adverse effects and recurrence were evaluated.

**Results:**

Sixty‐five percent of the ears exhibited an improvement of > 50% following treatment. On average, maximal lesion resolution occurred within 52.8 days (range 6–74 days). None of the horses exhibited severe sensitivity during the treatment period, allowing for ear manipulation throughout the study.

**Conclusions and Clinical Relevance:**

The topical application of 0.5% tretinoin cream proved to be an effective and safe treatment for equine aural plaques. Further studies with a larger sample size and long‐term follow‐up are recommended to optimise treatment protocols.

## Graphical abstract

Background: Equine aural plaques are a benign form of auricular papillomatosis caused by equine papillomavirus. Despite the efficacy of 5% imiquimod cream, a marked local reaction is frequently observed during therapy, often requiring sedation before application. Hypothesis/Objectives: The aim of this study was to evaluate the efficacy and potential adverse effects of 0.5% topical tretinoin in the treatment of equine aural plaques. We hypothesised that tretinoin would be effective, with fewer adverse effects compared to imiquimod. Conclusions and Clinical Relevance: The topical application of 0.5% tretinoin cream proved to be an effective and safe treatment for equine aural plaques. Further studies with a larger sample size and long‐term follow‐up are recommended to optimise treatment protocols.

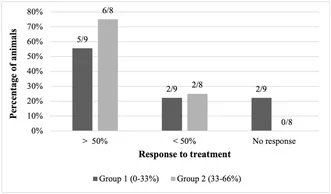

## 1 Introduction

Equine aural papillomatosis, also known as aural plaque, is a benign dermatological disease caused by equine papillomavirus (EcPV). This dermatopathy occurs on the inner surface of one or both ears of the horse, without spontaneous regression, and is usually painless. Although asymptomatic in most cases, increased sensitivity and discomfort in the ears, especially during manipulation and halter placement, may be observed [1, 2, 3]. Lesions may appear as isolated papules or plaques and can evolve into multiple coalescent, whitish, hyperkeratotic plaques that may extend to involve the entire auricular surface [2, 4].

Several types of *Equus caballus* papillomavirus (EcPV) have been identified to date (EcPV‐1 to EcPV‐9), with types EcPV‐1–7 being more thoroughly characterised [5, 6]. Among these, EcPV‐3, EcPV‐4, EcPV‐5 and EcPV‐6 are most commonly associated with equine aural plaques [1]. In Brazil, PCR‐based studies have identified EcPV‐3, EcPV‐4 and EcPV‐6 in aural plaque samples, frequently occurring as co‐infections [6]. EcPV‐1, typically linked to classical cutaneous papillomatosis, also has been detected, although consistently in combination with other EcPV types, limiting conclusions regarding its independent role in aural plaque pathogenesis. EcPV‐2 and EcPV‐7 were not identified in these Brazilian samples [6]. More recent data from the same geographical region further support these findings, reporting EcPV‐6 as the most prevalent type, followed by EcPV‐3, EcPV‐4 and EcPV‐5, with no detection of EcPV‐2, EcPV‐7, EcPV‐8 or EcPV‐9 [7].

Clinical diagnosis is based on the characteristic appearance of lesions, while histopathological examination reveals epidermal hyperplasia, mild papillomatosis associated with orthohyperkeratosis, areas of hypomelanosis or complete absence of melanin, koilocytosis and increased size and number of keratohyalin granules [2, 8]. To date the most effective treatment for equine aural plaque is 5% imiquimod cream, which modulates the immune response, leading to localised inflammation [9]. Despite its efficacy, this medication frequently induces an intense inflammatory response and high skin sensitivity, making frequent application challenging and sometimes requiring sedation [10].

Retinoids are natural or synthetic vitamin A derivatives used to treat various human dermatological conditions such as acne, rosacea, psoriasis and lupus erythematosus [11]. Topical retinoids have been shown to reduce lesion volume and alter the quality and quantity of the stratum corneum in cutaneous warts caused by human papillomavirus [12]. Tretinoin, a first‐generation synthetic retinoid, has been used in veterinary dermatology for the treatment of benign cutaneous tumours, with promising results [13]. Reported adverse effects of topical use in humans include skin irritation, dryness, scaling, pruritus and erythema [12]. Systemic administration in dogs has been associated with adverse effects such as keratoconjunctivitis sicca, increased hepatic enzyme levels, hypertriglyceridaemia and hypercholesterolaemia. However, these adverse effects are dose‐dependent, and despite their occurrence, the therapy is generally well‐tolerated [14].

To date, no published studies have evaluated the efficacy of tretinoin in treating equine aural plaques. This study aims to assess the efficacy and potential adverse effects of tretinoin 0.5% in equine aural plaques. This research is justified by the need for an effective treatment for equine aural plaques with fewer and milder side effects than those reported for 5% imiquimod.

## 2 Materials and Methods

### 2.1 Ethical Aspects

This study was approved by the Animal Ethics Committee of the ‘Federal University of Viçosa’ under protocol 34/2022. Written informed owner consent was obtained before the experiment.

### 2.2 Animals

The study included 10 Mangalarga Marchador horses (A1 to A10), of both sexes, aged between 7 months and 15 years (mean age 3.4), with a clinical diagnosis of bilateral aural papillomatosis and without prior treatment. All horses were considered healthy before the experiment based on a thorough physical examination, which included evaluation of attitude, appetite, body condition score, colour of mucous membranes, capillary refill time, heart rate, respiratory rate, heart and lung sounds, pulse quality, intestinal motility, palpation of lymph nodes, body temperature and faecal characteristics. Complete blood cell count (CBC) and chemistry profile were performed and considered within normal limits before the experiment.

Before the experiment the location, size and aspect of the aural plaque lesions were documented using digital photographs for comparative analysis throughout the study. Initially, three horses with bilateral aural plaque participated in a pilot study (A1–A3). The horses were kept in stalls without direct exposure to sunlight, and only one ear per horse was treated, allowing the contralateral ear to serve as an untreated control. The remaining seven horses (A4–A10) were privately owned and maintained under routine regional husbandry conditions, in paddocks with habitual natural sunlight exposure throughout the treatment period. In these horses, both ears were treated, totalling 17 treated ears in the study.

Before treatment, the lesion size was classified based on the percentage of the affected region in the concave pinna and categorised into three intervals: 0%–33% of the inner ear affected (Group 1), 33%–66% of the inner ear affected (Group 2), and 66%–100% of the inner ear affected (Group 3).

### 2.3 Biopsy Samples

Biopsy samples were collected on five distinct ears (from horses A1–A3 in the pilot study), before initiation of therapy. Although all three horses presented bilateral lesions, one ear had a lesion that was too small to allow safe and representative biopsy sampling; therefore, a total of five ears were sampled. No biopsies were collected on horses A4–A10, as they were privately owned and not part of the pilot study. Before the procedure, the horses were sedated with detomidine (0.015 mg/kg, intravenous), and a regional nerve block was performed using 2% lidocaine, targeting the internal and greater auricular nerves [15]. Tissue samples were collected from the centre of the lesion using a 4 mm or 6 mm sterile disposable biopsy punch. All samples were immediately fixed in 10% formalin and submitted for histopathological processing. Slides were stained with haematoxylin & eosin for light microscopy analysis. Owing to the limited biopsy sample size, only three of the five samples were placed in cryotubes, frozen at −80°C and analysed by PCR for the detection of EcPV‐1 to EcPV‐9 [7, 16].

### 2.4 Topical Treatment With 0.5% Tretinoin

Lesions were treated with a 0.5% compounded tretinoin cream. The formulation was prepared by a licensed compounding pharmacy. The concentration was selected based on anecdotal clinical reports suggesting limited efficacy of lower concentrations in equine aural plaques. A thin layer of the cream was applied topically once daily using gentle local massage. All applications were performed by the same veterinary surgeon to ensure procedural consistency. In cases where local inflammatory reactions were observed, the treatment frequency was adjusted to every other day. Applications were performed in the evening and continued until lesion stabilisation or resolution had occurred, as determined by the evaluating veterinary surgeon. Treatment duration varied according to individual response, and no interruptions occurred owing to adverse effects or lack of owner compliance. Horses A1–A3 were kept out of direct sunlight, while A4–A10 remained in paddocks with normal sun exposure.

### 2.5 Evaluation of Treatment Response

A daily monitoring sheet was completed for each horse throughout the study, documenting general health parameters, including attitude, appetite, mucous membrane colour, capillary refill time, heart rate, respiratory rate, body temperature, intestinal motility, changes in palpable lymph nodes and faecal production. Treatment response was assessed daily, with the presence of erythema, oedema, bleeding, crusting, pruritus and local sensitivity recorded in the monitoring sheet. Lesions were documented daily through digital photographs of both inner ears.

In the pilot phase (A1–A3), monitoring focused on local clinical response and treatment tolerance. In the subsequent study phase (A4–A10), systemic safety monitoring was expanded, and a CBC, biochemical analysis and Schirmer tear test were performed before the experiment and every 7 days during the treatment period. The biochemical analysis included serum concentrations of total protein, albumin, aspartate aminotransferase (AST), alkaline phosphatase (ALP), gamma‐glutamyl transferase (GGT), urea, creatinine, cholesterol and triglycerides.

Behavioural signs evaluated included restlessness, frequent head shaking, ear tilting or backward positioning and pain responses upon touch or manipulation, using a scale adapted from a previous study [10] (Table 1). The signs were assessed based on their presence, intensity and duration. The total number of treatments and the total treatment duration for each ear were recorded. Lesion response to treatment was classified into three categories: those showing a > 50% reduction in size (> 50% improvement); those showing improvement with < 50% reduction in size (< 50% improvement); and those with no discernible improvement. Following the treatment period, the horses were monitored for recurrence according to the following schedule: weekly during the first month, biweekly during the second month and monthly during the third month.

**TABLE 1:** Local sensitivity assessment scale (adapted from Zakia et al. [16]).

Sensitivity level	Description
Absent	No reaction to palpation
Mild	Mild response observed, without resistance to palpation
Moderate	More pronounced pain response; allows palpation only under routine physical restraint
Severe	Exaggerated reaction to palpation or prevents palpation of the ear

All lesion assessments were performed by the same veterinary surgeon with residency training in large animal internal medicine and clinical experience in equine dermatology. Lesion progression was independently reviewed by a board‐certified clinician (DACVIM) and by a full professor of veterinary dermatology with expertise in dermatopathology (certified specialist by the Brazilian Association of Veterinary Dermatology, ABDV). Interobserver agreement was 100%.

### 2.6 Statistical Analysis

All statistical analyses were performed using R software [17]. Plasma fibrinogen concentration was analysed as a categorical variable with four ordered levels (< 0.2, 0.2, 0.4 and 0.6). Associations between fibrinogen level and day of evaluation were assessed using contingency tables and Fisher's exact test. Treatment response was classified into three categories (no response, < 50% response and ≥ 50% response) and associations with day were analysed using contingency tables and Fisher's exact test. Schirmer's test measurements were compared between left and right eyes using paired Student's *t*‐tests. Continuous biochemical and haematological parameters were analysed to evaluate changes over time following treatment. Before model fitting, homoscedasticity was assessed using Bartlett's test, and error normality was evaluated using the Shapiro–Wilk test. For variables meeting model assumptions, generalised least squares (GLS) models were fitted, with day included as a fixed effect and animal as the repeated‐measures unit. To account for within‐animal correlation over time, two correlation structures were evaluated: compound symmetry and first‐order autoregressive. Model selection was based on Akaike's information criterion (AIC), and the correlation structure with the smallest AIC was retained for inference. The general model was specified as:

$$Y_{\mathrm{ij}=}\;\mu +D_{i}+e_{\mathrm{ij}}$$

where *Y*_*ij*_ represents the observed response for animal *j* on day *i*, μ is the overall constant, *D*_*i*_ is the fixed effect of day and *e*_*ij*_ is the random error term with a correlation structure defined within animals. *Post hoc* pairwise comparisons between days were conducted using estimated marginal means with Tukey's adjustment for multiple comparisons. For variables analysed using GLS models, data transformations were applied solely to satisfy model assumptions. AST values were inverse‐transformed (*y*´ = 1/*y*), while triglyceride concentrations and basophil counts were log‐transformed (*y*´ = log (*y*)). For clarity and biological interpretability, results are presented on the original measurement scale. When variables did not meet assumptions of normality or homoscedasticity, even after transformation, nonparametric analyses were performed using the Kruskal–Wallis test followed by Dunn's *post hoc* test. The significance level was set at α = 0.05 for all analyses.

## 3 Results

### 3.1 Histopathological Findings

Histopathological analysis revealed epidermal hyperplasia associated with mild papillomatosis, widened epidermal ridges and the presence of koilocytosis, along with a significant reduction in melanin pigmentation. Additionally, hyperkeratosis and hypergranulosis were consistently observed across all samples.

### 3.2 PCR

PCR analysis revealed the presence of EcPV‐4 in horse A1, co‐occurrence of EcPV‐1 and EcPV‐6 in horse A2 and EcPV‐3 in horse A3.

### 3.3 Physical Examination, CBC, Biochemical Analysis and Schirmer Tear Test

No changes in physical examination parameters and appetite were observed during the experiment. CBC results remained within the reference range for the species. Biochemical analysis revealed normal concentration of liver enzymes, total protein, albumin, cholesterol, triglycerides, creatinine and urea during the monitoring period. The Schirmer tear test, performed weekly, showed no significant changes in tear production during topical therapy with 0.5% tretinoin cream.

### 3.4 Lesion Assessment and Treatment Response

#### 3.4.1 Lesion Classification

At baseline (before treatment initiation), nine of the 17 treated ears (53%) were classified in Group 1 (0%–33% of the concave pinna affected) and eight ears (47%) were classified in Group 2 (33%–66% of the concave pinna affected). No lesions were classified as affecting > 66% of the concave pinna area.

### 3.5 Response to Treatment, Ear Sensitivity and Treatment Duration

**FIGURE 1:**
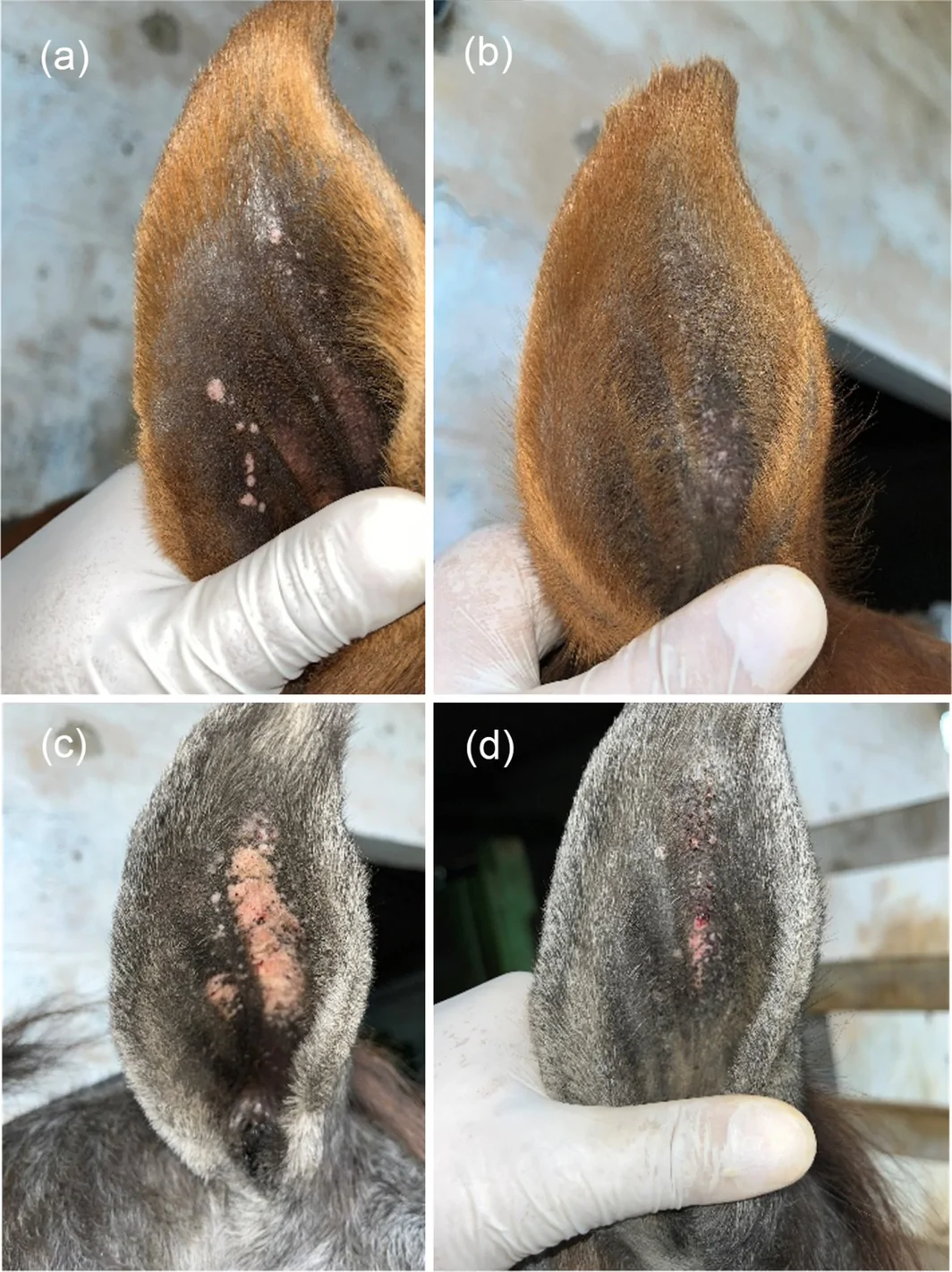
Ears before and after treatment, showing > 50% clinical improvement. (a) Baseline appearance before treatment; (b) same animal as in (a) after 6 days of treatment (six applications); (c) baseline appearance before treatment; and (d) same animal as in (c) after 56 days of treatment (41 applications)

**FIGURE 2:**
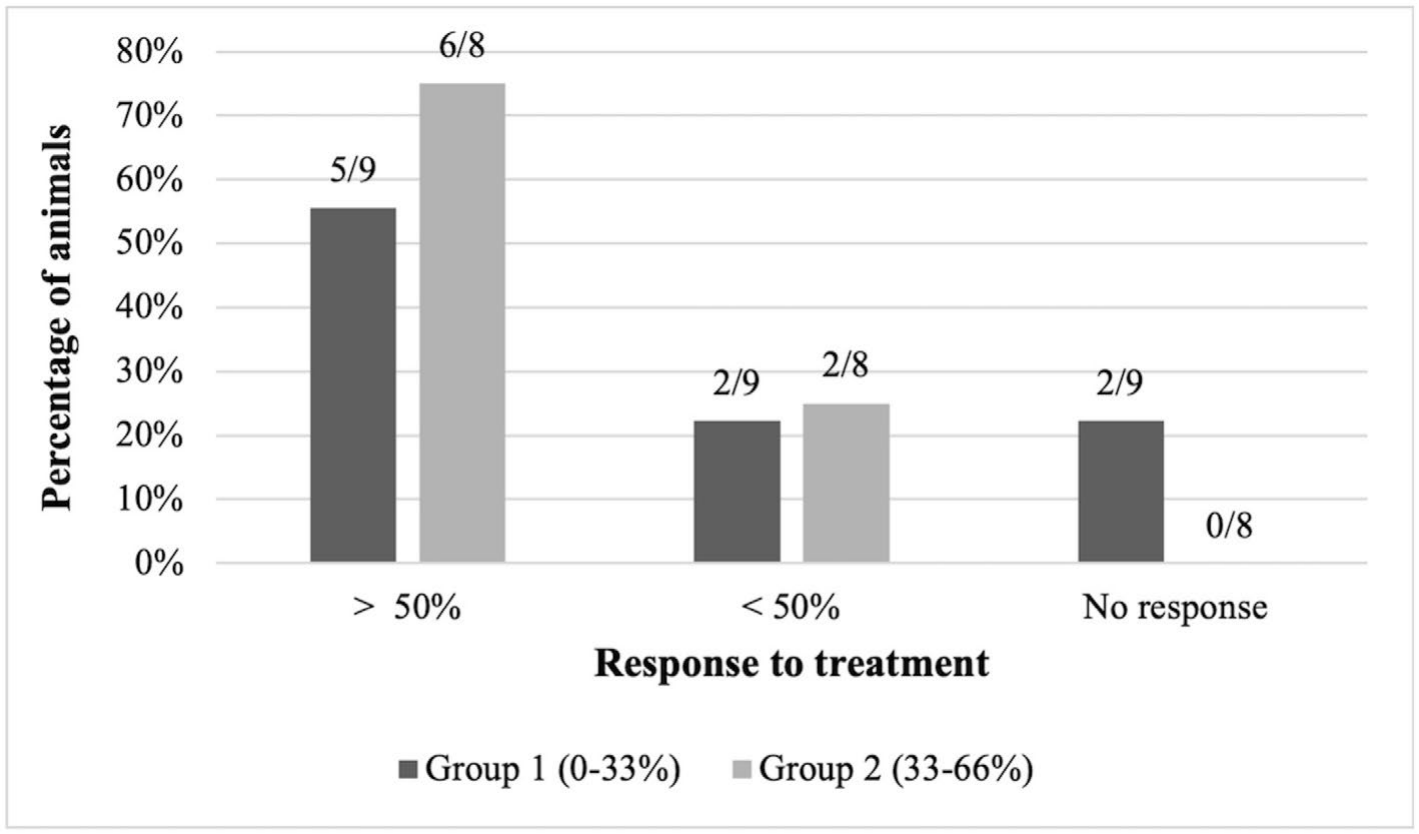
Association between initial lesion extent and treatment response in 10 horses (17 treated ears). *p* = 0.620 by Fisher's exact test.

Eleven of the 17 treated ears (65%) exhibited an improvement of > 50% following treatment (Figure 1), while four ears (24%) showed improvement with < 50% reduction. Only two ears (12%), both from the same horse, showed no improvement. Among the eight lesions classified as affecting > 33% of the concave pinna area, six demonstrated improvement > 50%. Five of the nine ears with < 33% of the concave pinna affected also improved by > 50% (Figure 2). Eight of the 17 (47%) treated ears showed an improvement of nearly 100%.

The mean treatment duration was 22 days for horses included in the pilot study (A1–A3; range 20–27 days) and 59 days for horses A4–A10 (range 6–74 days). On average, maximal lesion resolution occurred within 52.8 days (range 6–74 days). The number of applications of 0.5% tretinoin cream varied from 6 to 54 (13–18 in the pilot study and 6–54 in horses A4–A10), with a mean of 37 applications (15.6 in the pilot study and 41.6 in horses A4–A10).

Although no statistical analysis was performed owing to the small sample size, horses exposed to sunlight throughout the treatment period (A4–A10) demonstrated greater local sensitivity, required more tretinoin applications and had longer treatment durations on average compared to the pilot study animals (A1–A3).

None of the horses exhibited behavioural changes such as restlessness, head shaking, ear tilting or backward positioning. One horse showed no signs of local sensitivity during the experiment, four showed mild signs and five showed moderate signs; however, in these cases, restraint was limited to routine physical handling for palpation, and no additional restraint measures or sedation were required. The mean duration of sensitivity, at any level (mild or moderate), was approximately 43 days. Ear sensitivity lasted an average of 7 days in the pilot study horses, compared with an average of 51 days in horses A4–A10. All horses developed mild crusting and erythema between Day (D)3 and D13 of therapy. All except one horse showed mild ulceration. None of the horses exhibited oedema or pruritus. Lesions were monitored by digital photography for ≤ 124 days post‐treatment, with an average follow‐up period of approximately 71 days.

**FIGURE 3:**
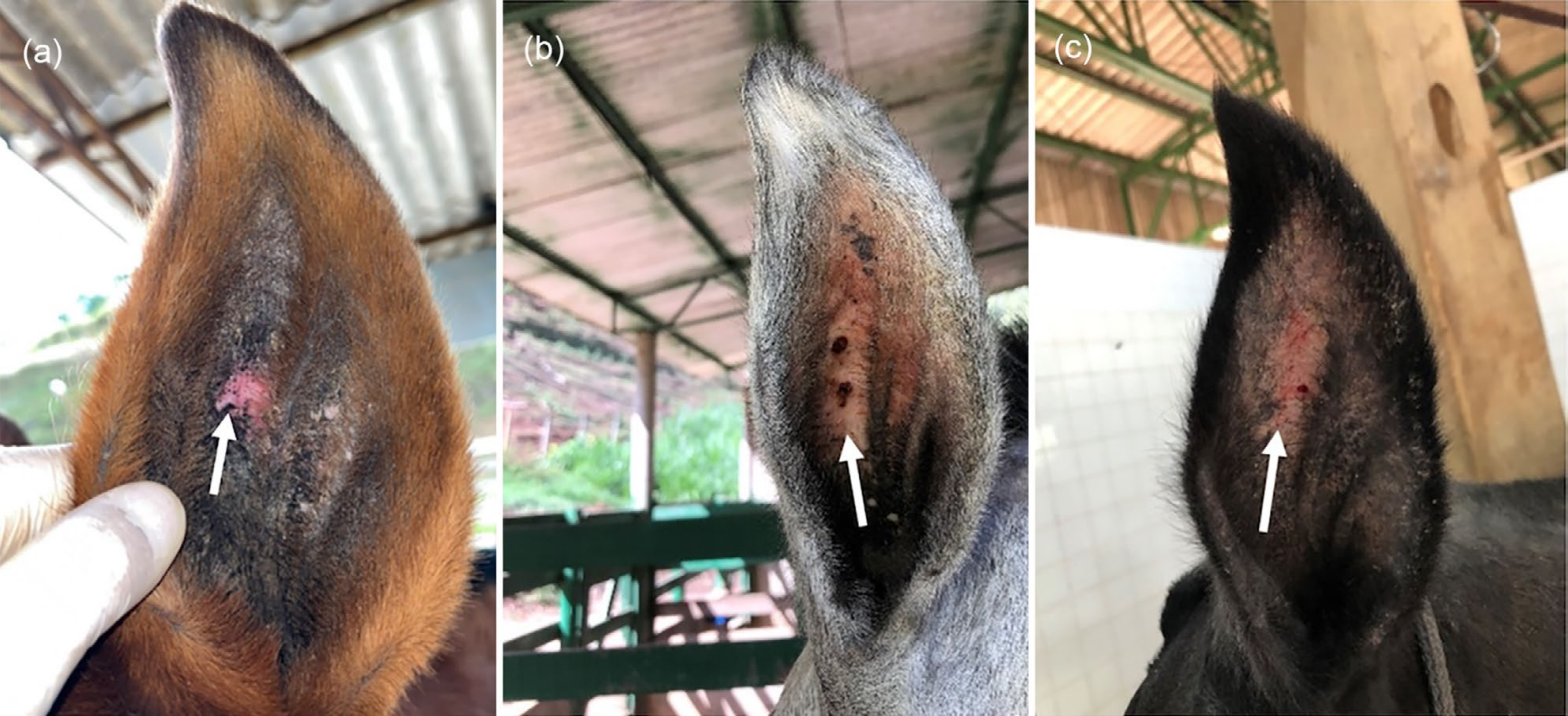
Post‐treatment erythema and hypopigmentation (white arrows). (a) Moderate erythema after 52 days of therapy, (b) hypopigmentation after 20 days of therapy and (c) mild erythema after 20 days of therapy.

Among the 17 evaluated ears, six (35%) showed recurrence during the post‐treatment monitoring period. No significant association was found between initial lesion severity and response to 0.5% tretinoin treatment (*p* = 0.620) or between initial lesion severity and presence of recurrence. However, a significant association was observed between recurrence and treatment response (*p* = 0.005), indicating that lesions with no recurrence were more likely to have responded with > 50% improvement. At the end of therapy, focal hypopigmentation and erythema were observed in 16 of the 17 treated lesions and persisted during the follow‐up period (Figure 3).

## 4 Discussion

The histopathological findings of this study align with those reported previously [2], which described the histopathological characteristics of equine aural plaques, including moderate‐to‐intense epidermal hyperplasia, mild papillomatosis and, in some sections, vacuolated cells resembling koilocytes. Similar histopathological alterations have been described in human warts caused by human papillomavirus, including papillomatosis, hyperkeratosis and the presence of koilocytes [18].

PCR analysis of pre‐treatment biopsy samples obtained from the pilot‐phase horses (A1–A3) confirmed the presence of EcPV‐1, EcPV‐3, EcPV‐4 and EcPV‐6, all previously associated with equine aural plaques in Brazil [6]. EcPV‐1 was detected in only one sample, in co‐infection with EcPV‐6, and its clinical importance in the development of aural plaques remains unclear. EcPV‐2 and EcPV‐7 were not detected in the samples analysed. Although based on a limited number of tested horses, our findings regarding co‐infections and EcPV type distribution are consistent with earlier Brazilian reports [6, 7].

No clinically relevant changes were observed in CBC and chemistry profile throughout the treatment period, suggesting that the topical administration of 0.5% tretinoin cream did not induce systemic adverse effects affecting haematological parameters, renal function, hepatic function and lipid metabolism. Likewise, the Schirmer tear test revealed no reduction in tear production, indicating that topical tretinoin did not lead to keratoconjunctivitis sicca in horses, a condition reported in dogs and humans following systemic isotretinoin therapy [14, 19].

Previous studies evaluated the efficacy of 5% imiquimod for treating aural plaques and reported that 10 of 16 horses required sedation before therapy as a consequence of the inflammatory response induced by the medication [20]. Likewise, another study found that 75% of horses treated with 5% imiquimod needed sedation to allow manipulation of the affected area and removal of treatment‐associated crusts owing to pain [10]. By contrast, in the present study, no horses exhibited sufficiently severe sensitivity to impede palpation or necessitate sedation before therapy. Reported adverse effects of 5% imiquimod include erythema, oedema, erosion, ulceration, crust formation and exudation [10, 20]. Of these, only crusting, erythema and ulceration were observed in the present study, and at a lower intensity than described previously for imiquimod, suggesting that topical treatment with 0.5% tretinoin was well‐tolerated.

The greater local sensitivity observed in horses exposed to sunlight suggests that environmental factors may influence cutaneous response to topical tretinoin. Additionally, these animals were younger, raising the possibility that age‐related differences in epidermal barrier function or inflammatory responsiveness could modulate sensitivity to retinoids. However, these associations remain speculative and should be interpreted cautiously owing to the limited sample size. Sunlight exposure is a well‐documented concern during topical tretinoin therapy in humans, primarily owing to increased photosensitivity and the risk of phototoxic reactions associated with retinoid‐induced thinning and inflammation of the superficial epidermis [19]. In the present study, despite evening application, some horses were naturally exposed to sunlight as part of routine management practices. Importantly, sensitivity remained mild and did not interfere with handling. Attempts to use UV‐protective ear covers were unsuccessful, as the horses consistently removed them.

Of the 17 treated ears, 11 (65%) demonstrated a therapeutic response defined as > 50% lesion improvement, while four ears (24%) showed improvement with < 50% reduction. Reported response rates for topical imiquimod in equine aural plaques range from approximately 87% to > 90% resolution [10, 20]. Although direct comparison is limited by differences in study design and outcome definitions, topical tretinoin may represent a well‐tolerated alternative. The two ears that showed no improvement were from the same horse, suggesting the possibility of individual variation in therapeutic response.

The focal hypopigmentation observed in 16 lesions at the end of therapy with 0.5% tretinoin also was reported in four of 16 horses treated with 5% imiquimod [20] and in two of eight horses in another study [10]. The occurrence of hypopigmentation may be associated with the cytopathic effect of EcPV on keratinocytes and melanocytes, leading to decreased melanin pigmentation. Another possible explanation is the inflammatory response triggered by 0.5% tretinoin, which may cause melanocyte damage, reducing melanin production and consequently altering skin pigmentation (post‐inflammatory hypopigmentation).

The recurrence rate in this study was 35%, and a statistically significant association was found between recurrence and treatment response, with non‐recurrent lesions showing a greater likelihood of > 50% improvement. This reinforces the importance of early and effective therapeutic response in reducing the likelihood of lesion recurrence. It is also possible that some of the recurrent cases observed in this study may not represent true relapses, and rather reinfections, as the horses were housed in collective paddocks, which may facilitate viral transmission through direct contact or mechanical vectors such as flies [1, 4].

This study has limitations that should be acknowledged. The sample size was small, particularly in the pilot phase, limiting statistical power and precluding more robust subgroup analyses. The study was not randomised, and management conditions differed between groups, including age distribution and sunlight exposure, which may have influenced treatment response. Despite these limitations, the findings demonstrate a consistent clinical response and provide evidence supporting the safety and efficacy of 0.5% topical tretinoin for equine aural plaques.

## 5 Conclusion

In this study population, topical 0.5% tretinoin cream resulted in > 50% clinical improvement in 15 of 17 treated ears, with only mild and manageable local sensitivity. No systemic adverse effects were detected. Compared to previously reported therapies such as topical imiquimod, local reactions appeared less severe in our cohort. These findings suggest that 0.5% topical tretinoin represents a safe and effective therapeutic option for equine aural plaques. Controlled studies with larger sample sizes are necessary to optimise treatment protocols.

## Author Contributions

**Yame Fabres Robaina Sancler‐Silva:** resources, investigation. **Fernanda Campos Mansur:** investigation, data curation. **Lissandro Gonçalves Conceição:** conceptualization, methodology, supervision, project administration, writing – review and editing. **Ytalo Galinari Henrique Schuartz:** data curation, investigation. **Cristian Silva Teixeira:** investigation, resources. **Cintia Fernandes Fidélis:** validation, resources. **Felipe Sperandio de Mattos:** data curation, investigation, formal analysis, visualization, writing – original draft. **Vitória Régia Melo Silva:** data curation, investigation, writing – original draft. **Alexandre Secorun Borges:** resources, methodology. **Jose Paes Oliveira‐Filho:** methodology, resources. **Cristiana Raach Bromberger:** resources, visualization. **Raffaella Bertoni Cavalcanti Teixeira:** conceptualization, methodology, validation, formal analysis, supervision, funding acquisition, project administration, writing – review and editing. **Marcel Ferreira Bastos Avanza:** visualization, resources. **José Ricardo Barboza Silva:** formal analysis, writing – review and editing. **José Dantas Ribeiro Filho:** validation, writing – review and editing, resources.

## Funding

This work was supported by Coordenação de Aperfeiçoamento de Pessoal de Nível Superior, Finance Code 001. Fundação de Amparo à Pesquisa do Estado de Minas Gerais. Conselho Nacional de Desenvolvimento Científico e Tecnológico.

## Conflicts of Interest

The authors declare no conflicts of interest.

## Data Availability

The data that support the findings of this study are available on request from the corresponding author. The data are not publicly available due to privacy or ethical restrictions.
